# Role of autoantibodies targeting interferon type 1 in COVID-19 severity: A systematic review and meta-analysis

**DOI:** 10.1016/j.jtauto.2023.100219

**Published:** 2023-10-14

**Authors:** Abolfazl Akbari, Alireza Hadizadeh, Mahdi Amiri, Neshat Najaf Najafi, Zahra Shahriari, Tannaz Jamialahmadi, Amirhossein Sahebkar

**Affiliations:** a Student Research Committee, Faculty of Medicine, Mashhad University of Medical Sciences, Mashhad, Iran; b School of Medicine, Tehran University of Medical Sciences, Tehran, Iran; c Research Center for Advanced Technologies in Cardiovascular Medicine, Cardiovascular Research Institute, Tehran University of Medical Sciences, Tehran, Iran; d Applied Biomedical Research Center, Mashhad University of Medical Sciences, Mashhad, Iran; e Biotechnology Research Center, Pharmaceutical Technology Institute, Mashhad University of Medical Sciences, Mashhad, Iran

**Keywords:** Interferon, COVID-19, Autoantibody

## Abstract

**Introduction:**

Impairment of the type I interferon (IFN–I) signaling pathway is associated with increased severity of COVID-19 disease. Here we have undertaken a systematic review and meta = analysis on the association between the severity of COVID-19 and IFN-1 autoantibodies (AAbs; aIFN-1, aIFN-α, aIFN-ω, and aIFN-β).

**Methods:**

Four databases, including Medline [PubMed], Embase, Web of Science, and Scopus, were systematically searched to find papers on the role of aIFN-1 and its subtype AAbs in the severity of COVID-19 infection. Data on the prevalence of aIFN-1, aIFN-α, aIFN-ω, and aIFN-β were pooled using random- or fixed-effects models. Subgroup analysis was performed based on disease severity. Odds ratios (OR) for COVID-19 disease outcome, including length of hospital stay, ICU admission and death, were calculated in relation to positive or negative plasma IFN-1 AAbs.

**Results:**

A total of 33 studies with 13023 patients were included. The overall prevalence of circulating aIFN-1, aIFN-α, and aIFN-ω AAbs was 17.8 % [13.8, 22.8], 7.2 % [4.7, 10.9], and 4.4 % [2.1, 8.6], respectively, and the overall prevalence of neutralizing aIFN-1, aIFN-α, aIFN-ω, and aIFN-β AAbs was 7.1 % [4.9, 10.1], 7.5 % [5.9, 9.5], 8.0 % [5.7, 11.1] and 1.2 % [0.4, 3.5], respectively. Circulating aIFN-α (OR = 4.537 [2.247, 9.158]), neutralizing aIFN-α (O = 17.482 [8.899, 34.342]), and neutralizing aIFN-ω (OR = 12.529 [7.397, 21.222]) were significantly more frequent in critical/severe patients than in moderate/mild patients (p < 0.001 for all). Anti–IFN–1 was more common in male subjects (OR = 2.248 [1.366, 3.699], p = 0.001) and two COVID-19 outcomes including ICU admission (OR = 2.485 [1.409, 4.385], p = 0.002) and death (OR = 2.593 [1.199, 5.604], p = 0.015) occurred more frequently in patients with positive anti–IFN–1.

Conclusion: aIFN-1 and its subtypes AAbs are associated with severe and critical COVID-19 disease and may be a predictive marker for a poor prognosis, particularly in men.

## Highlights

• Autoantibodies against IFN-1 and its subtypes increase in COVID-19 patients.
• The presence of autoantibodies against IFN-1 and its subtypes are associated with more severe COVID -19 disease.
• Autoantibodies against IFN-1 and its subtypes may predict worse COVID-19 disease outcomes.
• Autoantibodies against IFN-1 were more prevalent among male patients.

## 1 Introduction

Severe acute respiratory syndrome coronavirus-2 (SARS-CoV-2) began spreading in Wuhan, China, in late December 2019 and has caused nearly 7 million deaths by May 4, 2023 [1]. SARS-CoV-2 infection has shown a broad spectrum of clinical presentations, ranging from asymptomatic to severe disease associated with multiple organ failure and death [2,3]. Impairment of antiviral mechanisms such as interferon type 1 (IFN-1) has been associated with the severity of several life-threatening respiratory infections [4], including influenza pneumonia [5] and COVID-19 [6]. IFN-1 belongs to a family of cytokines that trigger important innate immune defense mechanisms during viral infection [7]. IFN-1 comprises several subtypes: IFN-α, IFN-β, IFN-ω, IFN-ε, and several other subtypes that are structurally related and have a variety of biological functions, including protection against pathogens, immune-regulation, differentiation, and development [8]. Their deficiency can lead to viral spread, and their overproduction can cause excessive inflammation, leading to a poor clinical outcome. Impairment of the IFN-1 pathway can be caused either by inherited genetic defects or by the production of autoantibodies (AAbs) directed against IFN-1 [9]. Several studies have shown that SARS-CoV-2 induces a low and delayed IFN response in severe and critical patients [2,10,11]. Casanova and Anderson showed that inherited or autoimmune deficiencies of IFN-1 are responsible for 15 %–20 % of all critical COVID-19 patients [12]. Recently, Goncalves detected anti–IFN–1 (aIFN-1) AAbs in 18 % of critically ill COVID-19 patients, whereas these AAbs were not detected in any of the patients with mild disease [9].

Detection of IFN-1 AAbs in the population infected with the SARS-CoV-2 virus has been proposed as a predictive marker for unfavourable COVID-19 outcomes; therefore, to test this observation, we systematically reviewed the available work on the association between COVID-19 severity and IFN-1 AAbs in the serum of COVID -19 patients.

## 2 Material and methods

### 2.1 Search strategy and study selection

This systematic review and meta-analysis were conducted in compliance with PRISMA (Preferred Reporting Items for Systematic Reviews and Meta-Analyses) protocols [13]. The initial search strategy was developed in PubMed and PubMed's Medical Subject Headings (MeSH) and applied to each database searched. We identified articles by searching PubMed, Scopus, Embase, and Web of Science through May 10, 2023, using the following search terms: “COVID-19″ and “autoantibody”. A complete list of search strategies for all databases is provided in Table S1. Studies reporting aIFN AAbs in patients with COVID-19 were extracted. We also searched Google Scholar and the reference lists of the included articles to identify further studies. We included only studies in English.

### 2.2 Inclusion and exclusion criteria

All included papers met the following criteria [1]: subjects being diagnosed COVID-19 based on a reverse transcription polymerase chain reaction (RT-PCR), serological tests or typical symptoms [2] at least one of the aIFN-1 or its subtypes AAbs had to have been measured. We excluded all case reports [14].

### 2.3 Eligibility and quality assessment

Four reviewers (A.A., M.A., N.N., and Z.S.) participated in selecting studies based on the articles' title, abstract and full text. In cases where agreement could not be reached, a fifth reviewer (A.S.) reviewed the eligibility to determine final inclusion. Two reviewers (M.A. and N.N.) independently reviewed included articles for quality. Included studies were assessed using the Joanna Briggs Institute (JBI) assessment tools, which provide an assessment tool for most study types, including observational studies [15]. Disagreements were resolved by discussion between the investigators or a third reviewer (A.S.).

### 2.4 Data extraction

Study characteristics were extracted from the included articles, including first author's last name, study conduct date, title, study design, study location (country), patient demographics, COVID-19 confirmatory method, IFN autoantibody assay, and history of systemic autoimmune rheumatic disease. The number of individuals in whom circulating or neutralizing aIFN-1/α/ω/β was detected was also recorded. In addition, characteristics of COVID-19 patients in studies comparing seropositive patients with seronegative patients to IFN-1 or its subtypes, including duration of admission, demographics, intensive care unit (ICU) admission, and mortality rate, were extracted. Data were stored in a Microsoft Excel spreadsheet (Redmond, WA). Data was collected on the plasma AABs levels in COVID-19 and non-COVID-19 patients or within COVID-19 patients according to disease severity.

### 2.5 Statistical analysis

The weighted pooled prevalence and 95 % confidence intervals (CIs) of aIFN-1, aIFN-α, aIFN-ω, and aIFN-β were calculated. The magnitudes of the estimated effects of AAbs on COVID-19 outcomes and the prevalence of AAbs in male patients were expressed as odds ratios (OR), with corresponding 95 % CIs in brackets. The prevalence of AAbs in critical/severe or moderate/mild groups was compared using OR and 95 % CIs. Sensitivity analysis was done after removing a study conducted on children [16]. Median values were converted to means using a formula described by Wan et al. [17]. p < 0.05 was considered statistically significant. Heterogeneity was calculated quantitatively using the I2 statistical test. If heterogeneity was high (Cochran's Q < 0.05), the random-effects model was used; otherwise, the fixed-effects model was used. Potential publication bias was examined using funnel plots [18]. All statistical analyses were done using Comprehensive Meta-Analysis Software (CMA v.3, Biostat Inc., Englewood, New Jersey, USA).

## 3 Results

**Fig. 1:**
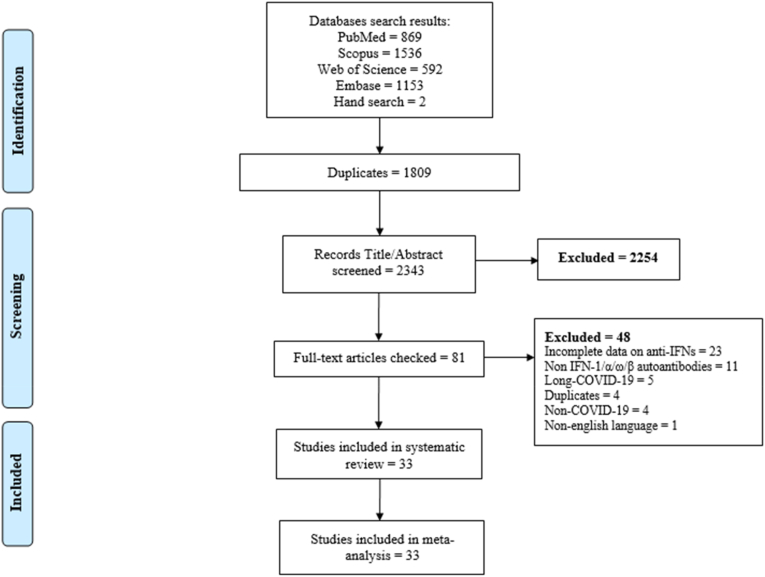
PRISMA flow diagram.

After removing 1809 duplicate papers, a total of 2343 papers were identified (Fig. 1). Finally, 33 studies were included in the present systematic review and meta-analysis. Four studies were suspected to be the same patients and were removed [[19], [20], [21], [22]]. The selected studies and their characteristics are listed in Table 1. Seventeen studies were conducted in European countries (France = 7 [9,[23], [24], [25], [26], [27], [28]], Spain = 3 [11,29,30], Italy = 3 [6,10,31], Netherlands = 2 [32,33], Belgium = 1 [34] and Switzerland = 1 [35]), six of the selected studies were conducted in North America (United States [7,[36], [37], [38], [39], [40]]) and one in South America (Colombia [41]), three in Asia (Iran = 1 [42], Russia = 1 [43], and Japan = 1 [44]) and six studies were multicenter studies [16,19,22,[45], [46], [47]]. Details of the quality assessment are reported in Table S2.

**Table 1:** Characteristics of the included studies.

First author (year/country)	Study design	Sample size (number and critical situation)	Age (mean ± SD)	Male	Auto-antibody (assay)	History of systemic autoimmune rheumatic disease	PCR for SARS-CoV-2
Goncalves et al. (2020/France)	Prospective cohort	84 critical (admitted to ICU) and 10 mild*	65.4 ± 11.6	67	aIFN-α2, aIFN-ω, IFN-β (ELISA)	9 autoimmune disease	Yes
Wijst et al. (2020/United States)	Prospective cohort	284 (26 critical, 102 severe, 156 moderate)*	51.9 ± 16.2	195	aIFN-α2 (radioligand binding assay)	ND	Yes
Solanich et al. (2021/Spain)	Retrospective	275 severe admitted to ICU*	63.3 ± 11.9	211	aIFN-α2, aIFN-ω (ELISA)	No	Yes
Chauvineau-Grenier (2020/France)	Prospective cohort	139 critical*	64.4 ± 15.8	86	aIFN-α2, aIFN-ω, aIFN-β (luciferase reporter assay, ELISA)	No	ND
Bastard et al. (2020/Multicenter)	Prospective cohort	987 critical, 663 asymptomatic or mild*	ND	761 males among critically ill patients	aIFN-α2, aIFN-ω (ELISA)	No	Yes
Bastard et al. (2020/Multicenter)	Prospective cohort	3595 critical, 623 severe, 1639 asymptomatic or paucisymptomatic*	ND	ND	aIFN-α2, aIFN-ω, aIFN-β (Multiplex particle-based assay, ELISA)	No	Yes (PCR and/or serological test and/or displaying typical symptom)
Matuozzo et al. (2023/Multicenter)	Cohort	928	ND	ND	aIFN-1	ND	Yes
Akbil et al. (2021/Multicenter)	Prospective cohort	430 (237 critical)*	62 ± 15.6	312	aIFN-α, aIFN- ω (ELISA)	14 autoimmune diseases	Yes
Abers et al. (2020/Italy)	Prospective cohort	218 (135 critical, 44 severe, 39 mild/moderate)	ND	ND	aIFN-α, aIFN-ω, aIFN-β (ELISA)	ND	Yes
Troya et al. (2020/Spain)	Cohort	31 severe and 16 critical	68.5 ± 4	28	aIFN-α2, aIFN-ω (luciferase reporter assays, ELISA)	No	Yes
Frasca et al. (2020/Italy)	Cohort	360*	62.25 ± 12.5	249	aIFN‐α, aIFN-ω, aIFN‐β **(**ELISA)	No	Yes
Lopez et al. (2021/France)	Prospective cohort	26 critical, 44 mild*	44.9 ± 10.0	24	aINF-α2 (ELISA)	ND	Yes
Troya et al. (2022/Spain)	Retrospective cohort	178 critical*	73.8 ± 3.8	115	aINF-α2, aIFN-ω, aIFN-β	19 autoimmune diseases	ND
Koning et al. (2020/Netherlands)	Prospective cohort	210 severe to critical	64.2 ± 5.1	133	aIFN-1 (multiplex particle-based assay, ELISA)	ND	Yes
Eto et al. (2022/Japanese)	Prospective cohort	627 (170 critical, 235 severe, 112 moderate, 105 mild, 5 asymptomatic)*	60.0 ± 20.1	440	aINF-α2, aIFN-ω (ELISA and ISRE reporter assays)	ND	ND
Raadsen et al. (2020/Netherlands)	Prospective cohort	282 (100 mild, 43 moderate, 97 severe, 38 critical)*	54.4 ± 15.7	229	aINF-α2 (ELISA and a pseudo virus–based neutralization assay)	ND	Yes
Chang et al. (2021/United States)	Prospective cohort	147*	57.4 ± 15.7	56	aIFN-1 (ELISA)	ND	Yes
Wang et al. (2020/United States)	Prospective cohort	194 (55 severe, 103 moderate, 7 mild, 29 asymptomatic)*	60.4 ± 18.7	87	aIFN-1 ELISA)	ND	Yes
Vazquez et al. (2020/United States)	Retrospective cohort	116 hospitalized*	ND	ND	aINF-α2 (radioligand binding assay), aIFN-ω (cell-based assay)	ND	Yes
Manry et al. (2022/France)	Cohort	1261 died	70.7 ± 13.0	821	aINF-α, aIFN-ω, aIFN-β (ELISA)	ND	ND
Savvateeva et al. (2021/Russia)	Cohort	86 critical*	ND	ND	aINF-α, aIFN-ω (microarray-based assay)	ND	Yes
Acosta-Ampudia et al. (2020/Colombia)	Cohort	19 recovered, 18 severe	50.7 ± 8.7	10	aINF-α2 (ELISA)	ND	Yes
Yee et al. (2021/United States)	Prospective cohort	103 inpatients and 24 outpatients	55.2 ± 15.2	86	aIFN-α, aIFN-ω	ND	ND
Steels et al. (2022/Belgium)	Prospective cohort	52 severe*	65.7 ± 11.8	38	aIFN-α2 (Luminex bead-based assay)	ND	Yes
Ziegler et al. (2022/United States)	Prospective cohort	8 severe and 12 mild or moderate*	ND	ND	aIFN-α2, aIFN-ω (microarray-based platform)	ND	Yes
Soltani-Zangbar et al. (2022/Iran)	Cross-sectional	50 severe and 50 mild*	46.7 ± 9.0	54	aIFN-α2 (ELISA)	No	Yes
Scordio et al. (2022/Italy)	Cohort	3 critical, 3 moderate, and 2 mild*	57.5 ± 13.2	7	aINF-α, aIFN-ω, aIFN-β (bioassay)	ND	Yes
Busnadiego et al. (2021/Switzerland)	Prospective cohort	103 critical*	64.3 ± 11.3	80	aINF-α2, aIFN-ω, aIFN-β (bead-based serological assay)	No	Yes
Mathian et al. (2022/France)	Retrospective cohort	5	ND	ND	aINF-α2, aIFN-ω, aIFN-β (ELISA)	5 systemic lupus erythematosus	ND
Smith et al. (2022/Multicenter)	Cohort	126*	ND	ND	aINF-α2, aIFN-ω (ELISA)	ND	Yes
Arrestier et al. (2022/France)	Prospective cohort	925 critical	61.6 ± 12.4	652	aINF-α2, aIFN-ω, aIFN-β (luciferase reporter assay)	ND	Yes
Bodansky et al. (2023/Multicenter)	Cohort	168 severe and 45 mild	11.1 ± 8.3	107	aINF-α2 (radioligand binding assay)	ND	Yes
Carapito et al. (2022/France)	Prospective cohort	47 critical and 25 non-critical	39.6 ± 9.8	53	aIFN-1 (ELISA)	No	Yes

aIFN: Anti-Interferon, ND: Not determined, ELISA: Enzyme-linked immunosorbent assay, PCR: Polymerase chain reaction*The study has healthy control group.

### 3.1 Circulating and neutralizing aIFN-1/α/ω/β

**Fig. 2:**
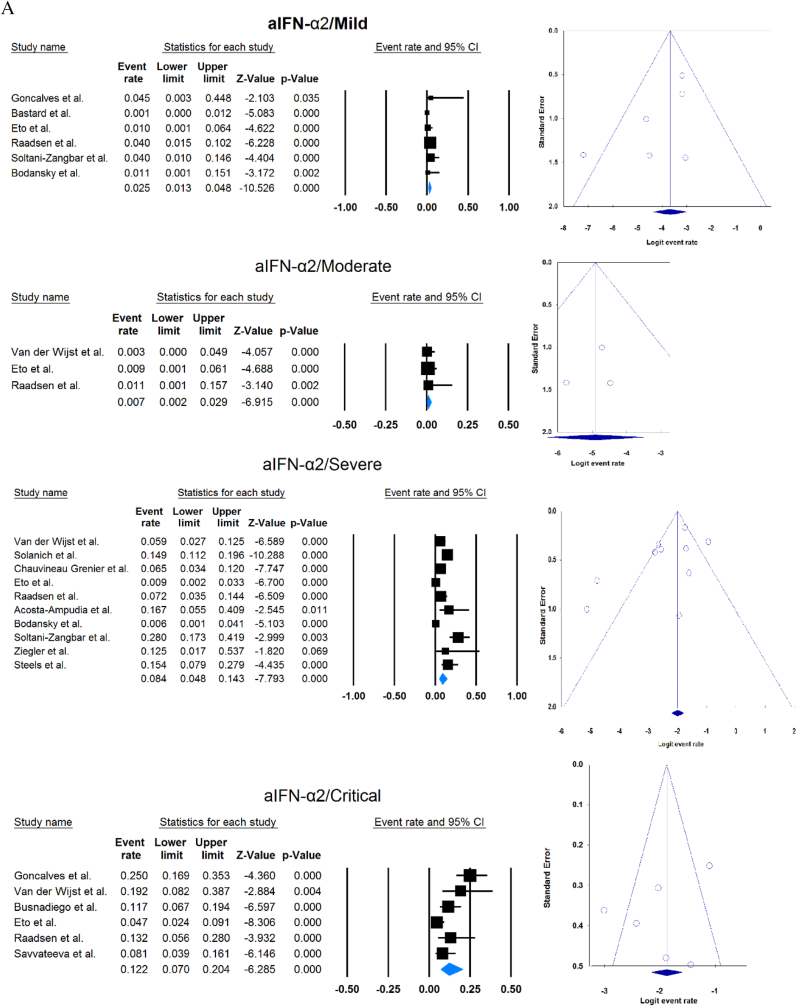

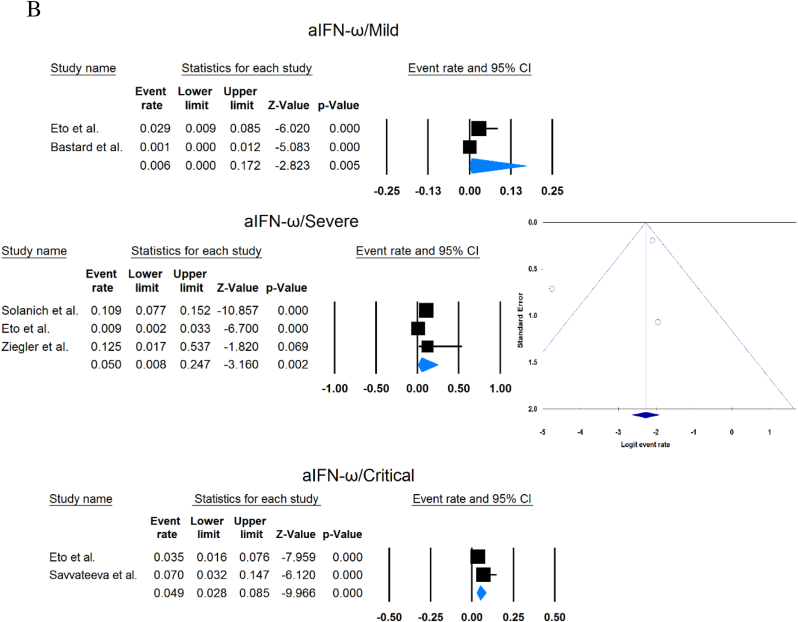
Prevalence of circulating autoantibodies against interferon α2 (A) and ω (B) in COVID -19 patients with different disease severity.

**Fig. 3:**
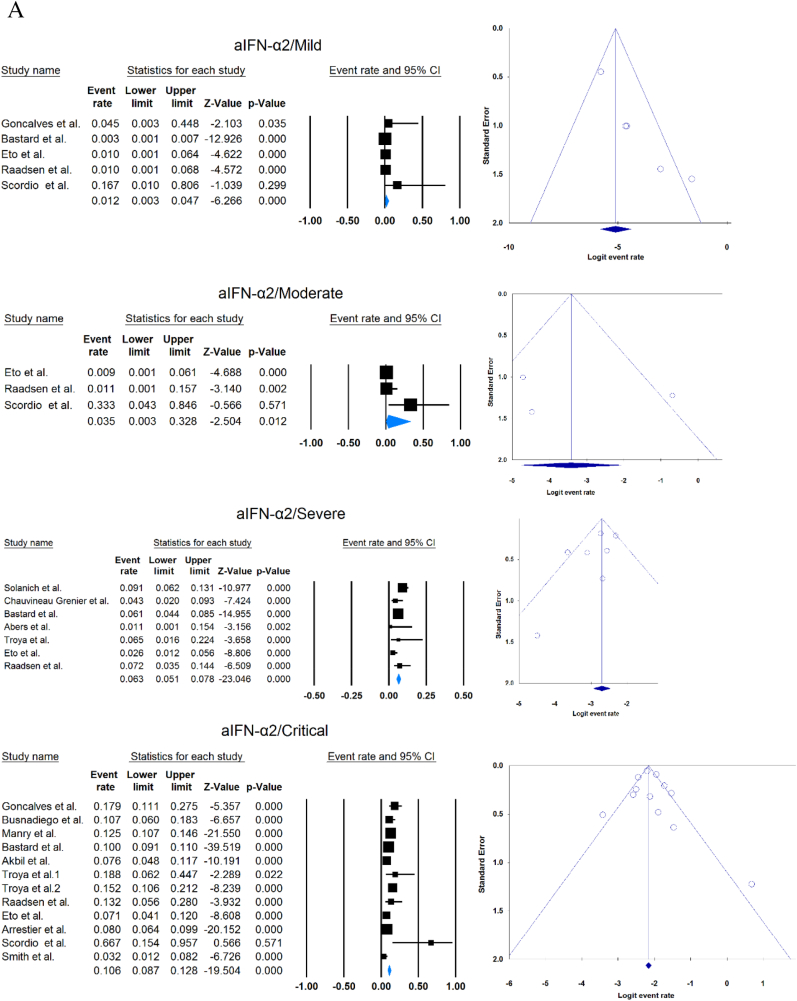

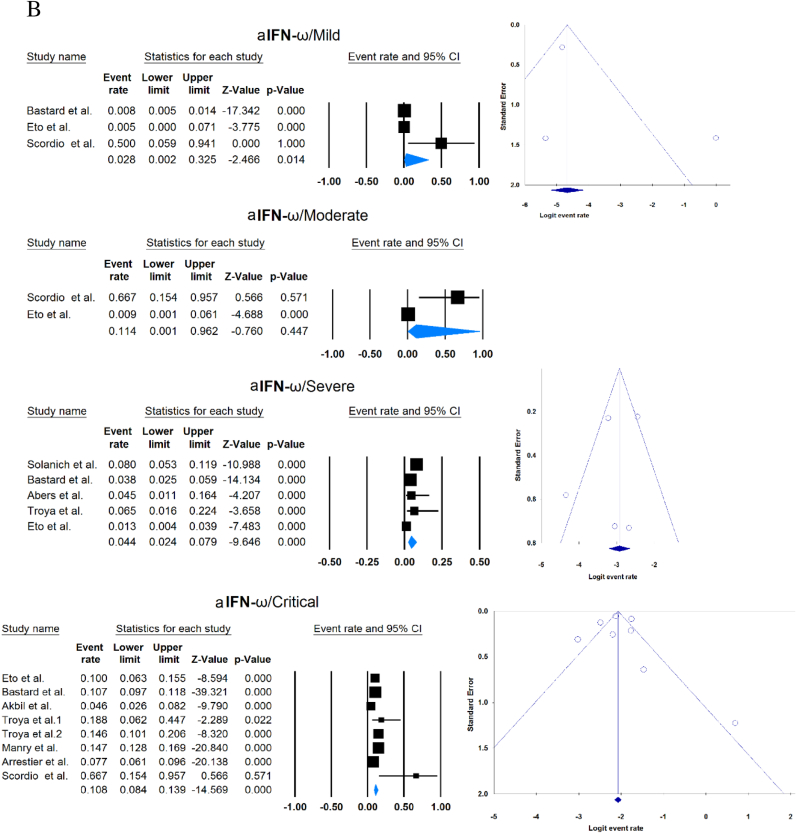

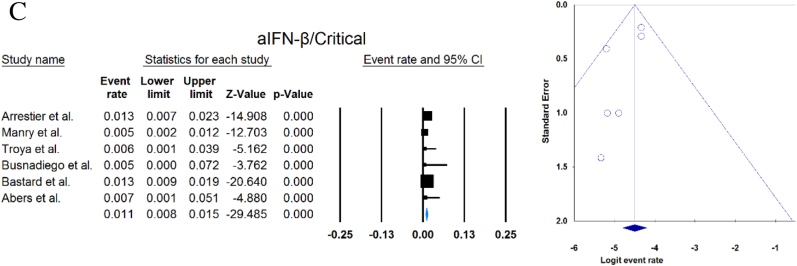
Prevalence of neutralizing autoantibodies against interferon α2 (A), ω (B), and β (C) among COVID -19 patients with different disease severity.

A total of 13023 patients (from 33 studies) diagnosed with COVID-19 and had at least one of the AAbs targeting IFN-1, IFN-α, IFN-ω, or IFN-β were included in this analysis. Seropositivity of patients with different stages of disease against IFN-1 and its subtypes is detailed in Table 2. Forest plots and funnel plots for each analysis are shown in Fig. 2, Fig. 3. The overall prevalence of circulating aIFN-1, aIFN-α, and aIFN-ω AAbs was 17.8 % [13.8, 22.8], 7.2 % [4.7, 10.9], and 4.4 % [2.1, 8.6], respectively, among COVID-19 patients, regardless of disease severity (Fig. 5). Sensitivity analysis by removing the study by Bodansky et al. showed 7.9 % [5.2, 11.8] prevalence of circulating aIFN-α. Also, the overall prevalence of neutralizing aIFN-1, aIFN-α, aIFN-ω, and aIFN-β AAbs was 7.1 % [4.9, 10.1], 7.5 % [5.9, 9.5], 8.0 % [5.7, 11.1], and 1.2 % [0.4, 3.5], respectively, regardless of disease severity (Fig. 6).

**Table 2:** Seropositivity of patients with different severity of disease regarding aIFN-1 and its subtypes AAbs.

First author	Sample size	aIFN-l- Circulating/Neutralizing	Circulating aIFN-α2/ω/β				Neutralizing activity against aIFN-α2/ω/β			
			Critical	Severe	Moderate	Mild	Critical	Severe	Moderate	Mild
Goncalves et al.	94	−/−	21 of 84/10 of 21*/1 of 21*	−/−/-	−/−/-	0 of 10/−/−	15 of 84/10 of 21*/1 of 21*	−/−/-	−/−/-	0 of 10/−/−
Van der Wijst et al.	284	−/−	5 of 26/−/−	6 of 102/−/−	0 of 156/−/−	−/−/-	−/−/-	−/−/-	−/−/-	−/−/-
Solanich et al.	275	49/26	−/−/-	41 of 275/30 of 275/-	−/−/-	−/−/-	−/−/-	25 of 275/22 of 275/-	−/−/-	−/−/-
Chauvineau-Grenier et al.	139	−/−	−/−/-	9 of 139/-/0 of 86	−/−/-	−/−/-	−/−/-	6 of 139/-/0 of 86	−/−/-	−/−/-
Busnadiego et al.	103	12/-	12 of 103/8 of 103/0 of 103	−/−/-	−/−/-	−/−/-	11 of 103/-/0 of 103	−/−/-	−/−/-	−/−/-
Bastard et al. (Duplicate)	1650	−/−	−/−/-	−/−/-	−/−/-	0 of 663/0 of 663/-	88 of 987/65 of 987/-	−/−/-	−/−/-	0 of 663/0 of 663/-
Bastard et al.	4117	-/523	−/−/-	−/−/-	−/−/-	−/−/-	360 of 3595/385 of 3595/23 of 1773	32 of 522/20 of 522/0 of 187	−/−/-	5 of 1639/13 of 1639/-
Akbil et al.	403	28/-	20 of 403/17 of 403/-				18 of 237/11 of 237/-	−/−/-	−/−/-	−/−/-
Abers et al.	44	2/2	−/−/-	−/−/-	−/−/-	−/−/-	−/−/1 of 135	0 of 44/2 of 44/-	−/−/-	−/−/-
Troya et al.	47	5/5	−/−/-	−/−/-	−/−/-	−/−/-	3 of 16/3 of 16/-	2 of 31/2 of 31/-	−/−/-	−/−/-
Frasca et al.	360	61/-	−/−/-	−/−/-	−/−/-	−/−/-	13 of 360/9 of 360/37 of 360			
Lopez et al.	56	−/−	10 of 56/−/−				−/−/-	−/−/-	−/−/-	−/−/-
Troya et al.	178	−/−	−/−/-	−/−/-	−/−/-	−/−/-	27 of 178/26 of 178/1 of 178	−/−/-	−/−/-	−/−/-
Koning et al.	210	35/6	−/−/-	−/−/-	−/−/-	−/−/-	−/−/-	−/−/-	−/−/-	−/−/-
Eto et al.	622	-/26	8 of 170/6 of 170/-	2 of 235/2 of 235/-	1 of 112/0 of 112/-	1 of 105/3 of 105/-	12 of 170/17 of 170/-	6 of 235/3 of 235/-	1 of 112/1 of 112/-	1 of 105/0 of 105/-
Raadsen et al.	282	−/−	5 of 38/−/−	7 of 97/−/−	0 of 43/−/−	4 of 100/−/−	5 of 38/−/−	7 of 97/−/−	0 of 43/−/−	1 of 100/−/−
Chang et al.	48	23/-	−/−/-	−/−/-	−/−/-	−/−/-	−/−/-	−/−/-	−/−/-	−/−/-
Wang et al.	194	52/-	−/−/-	−/−/-	−/−/-	−/−/-	−/−/-	−/−/-	−/−/-	−/−/-
Vazquez et al.	116	−/−	4 of 116/−/−				2 of 116/−/−			
Manry et al.	1121	305/-	−/−/-	−/−/-	−/−/-	−/−/-	140 of 1121/165 of 1121/6 of 1094	−/−/-	−/−/-	−/−/-
Savvateeva et al.	86	10/-	7 of 86/6 of 86/-	−/−/-	−/−/-	−/−/-	−/−/-	−/−/-	−/−/-	−/−/-
Yee et al.	127	-/4	−/−/-	−/−/-	−/−/-	−/−/-	4 of 103 (IFN-α or IFN-ω)		0 of 24 (IFN-α or IFN-ω)	
Steels et al.	52	−/−	−/−/-	8 of 52/−/−	−/−/-	−/−/-	−/−/-	−/−/-	−/−/-	−/−/-
Ziegler et al.	20	−/−	−/−/-	1 of 8/1 of 8/-	0 of 12/0 of 12/-	−/−/-	−/−/-	−/−/-	−/−/-	
Soltani-Zangbar et al.	100	−/−	−/−/-	14 of 50/−/−	−/−/-	2 of 50/−/−	−/−/-	−/−/-	−/−/-	−/−/-
Scordio et al.	8	−/−	−/−/-	−/−/-	−/−/-	−/−/-	2 of 3/2 of 3/-	−/−/-	1 of 3/2 of 3/-	0 of 2/1 of 2/-
Mathian et al.	5	−/−	−/−/-	−/−/-	−/−/-	−/−/-	4 of 5/4 of 5/2 of 5	−/−/-	−/−/-	
Smith et al.	126	−/−	−/−/-	−/−/-	−/−/-	−/−/-	4 of 126/−/−			
Arrestier et al.	925	-/96	−/−/-	−/−/-	−/−/-	−/−/-	74 of 925/71 of 925/12 of 925	−/−/-	−/−/-	−/−/-
Bodansky et al.	213	−/−	−/−/-	1 of 168/−/−	−/−/-	0 of 45/−/−	−/−/-	−/−/-	−/−/-	−/−/-
Matuozzo et al. (Duplicate)	928	234/-	−/−/-	−/−/-	−/−/-	−/−/-	−/−/-	−/−/-	−/−/-	−/−/-
Acosta-Ampudia et al.	18	−/−	−/−/-	3 of 18/−/−	−/−/-	−/−/-	−/−/-	−/−/-	−/−/-	−/−/-
Carapito et al.	72	2/-	−/−/-	−/−/-	−/−/-	−/−/-	−/−/-	−/−/-	−/−/-	−/−/-
**Total**	**13023**	**818/688**	**12.2 % [7.0, 20.4]/4.9 % [2.8, 8.5]/0 %**	**8.4 % [4.8, 14.3]/5.0 % [0.8, 24.7]/0 %**	**0.7 % [0.2, 2.9]/0 %/-**	**2.5 % [1.3, 4.8]/1.7 % [0.6, 4.7]/-**	**10.6 % [8.7, 12.8]/10.8 % [8.4, 13.9]/1.1 % [0.8, 1.5]**	**6.3 % [5.1, 7.8]/4.4 % [2.4, 7.9]/0 %**	**3.5 % [0.3, 32.8]/11.4 % [0.1, 96.2]/-**	**1.2 % [0.3, 4.7]/2.8 % [0.2, 32.5]/-**

aIFN: Anti-Interferon*Among patients who were positive for aIFN-α2.

Outcomes of COVID-19 in positive versus negative aIFN-1/α patients.

**Fig. 4:**
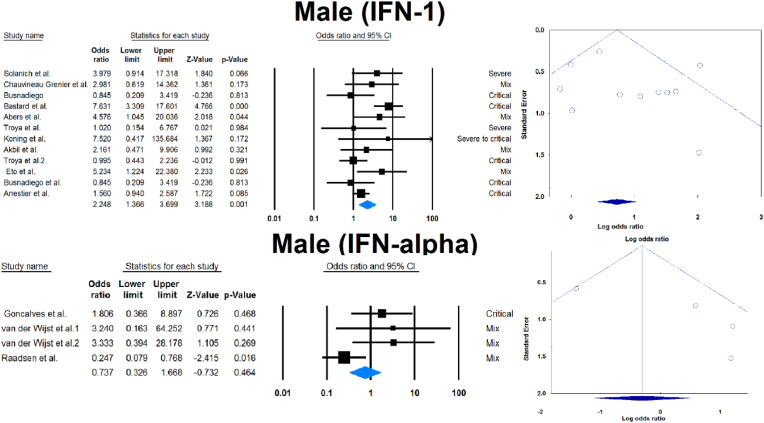

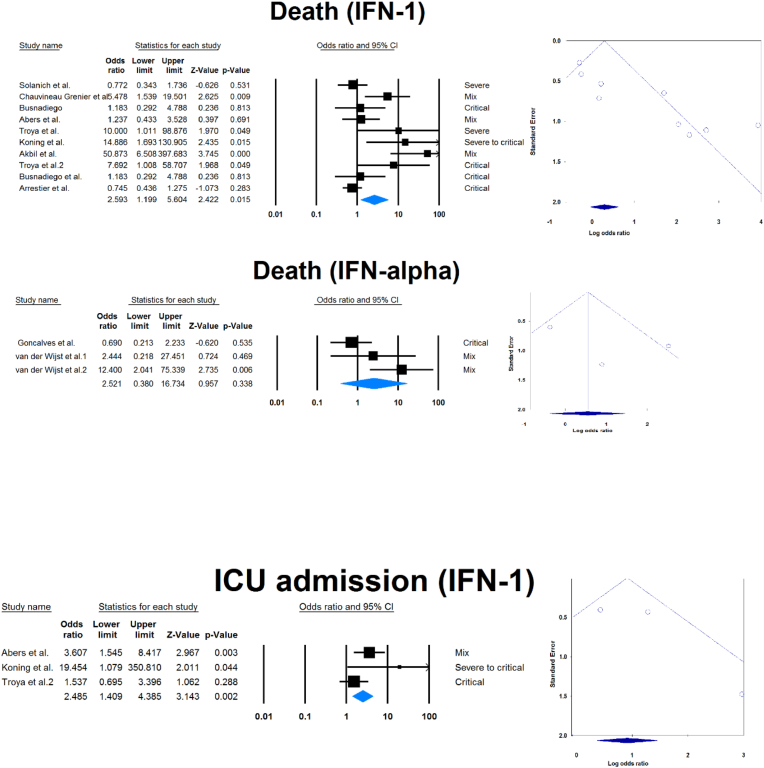

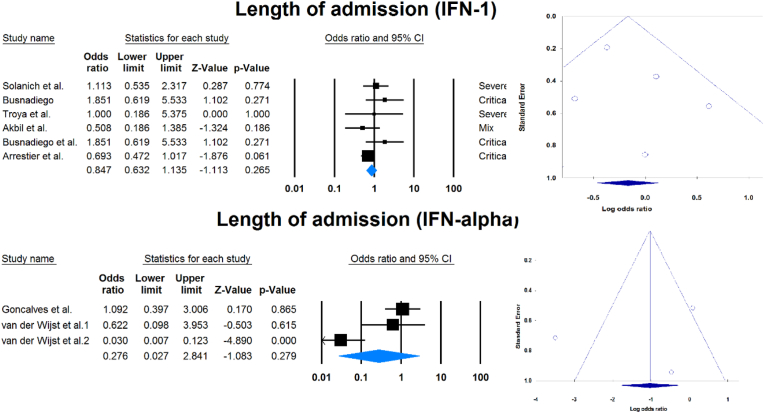
Association between sex, death, ICU admission, and length of hospital stay with positive serum autoantibodies against IFN-1 or IFN-α.

Fourteen studies compared patients with positive and negative AAbs to IFN-1 or IFN-α. Table 3 and Fig. 4 show the association between sex, death, ICU admission, and length of hospital stay with positive serum AAbs against IFN-1 or IFN-α.

**Table 3:** Comparison of COVID -19 results in seropositive patients and serunegative patients.

Variable	aIFN	Sample size (positive/negative)	OR [95 % CI]	P-value
Male	1	365/3917	2.248 [1.366, 3.699]	0.001
	α	39/612	0.737 [0.326, 1.668]	0.464
Death	1	238/2316	2.593 [1.199, 5.604]	0.015
	α	26/343	2.521 [0.380, 16.734]	0.338
ICU admission	1	63/514	2.485 [1.409, 4.385]	0.002
Length of admission	1	164/1692	0.847 [0.632, 1.135]	0.265
	α	26/343	0.276 [0.027, 2.841]	0.279

ICU: Intensive care unit, IFN: Interferon, OR: Odds ratio, CI: confidence interval.

**Fig. 5:**
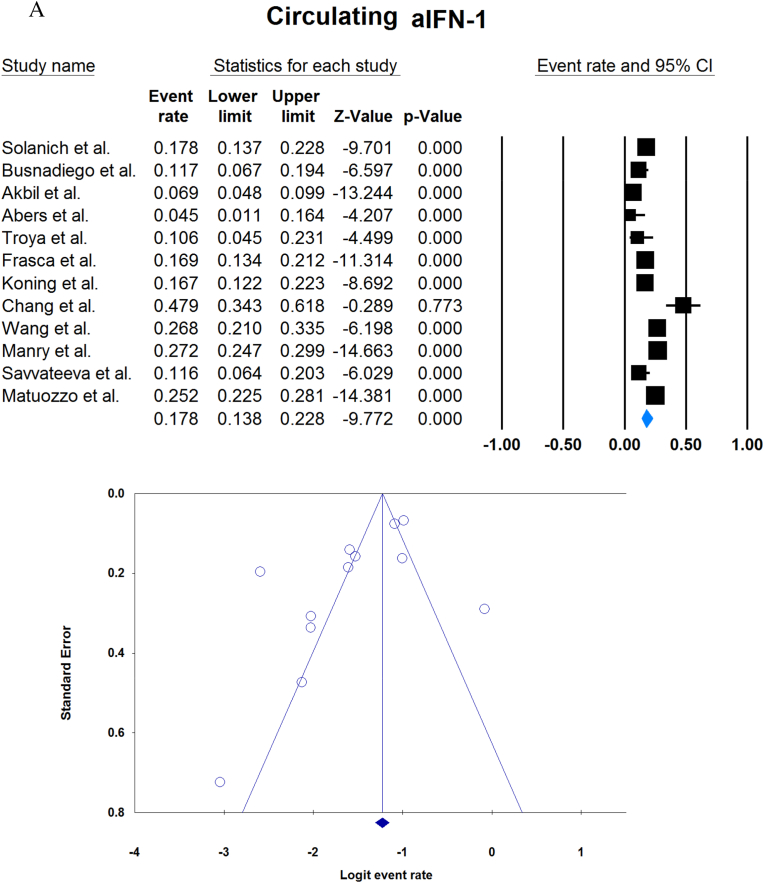

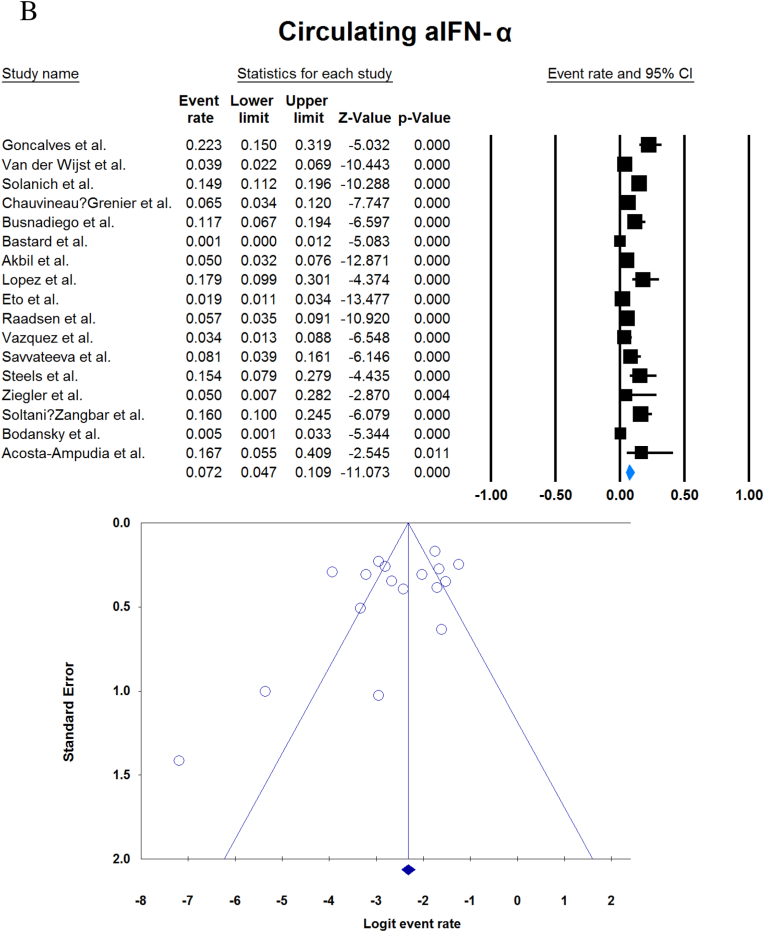

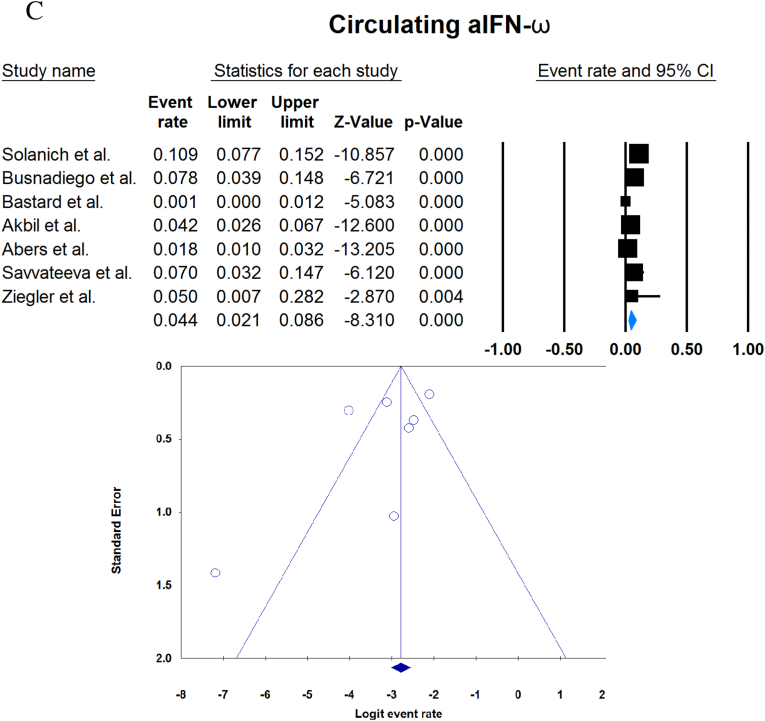
Overall prevalence of circulating aIFN-1 (A), aIFN-α (B), and aIFN-ω (C) autoantibodies regardless of disease severity.

**Fig. 6:**
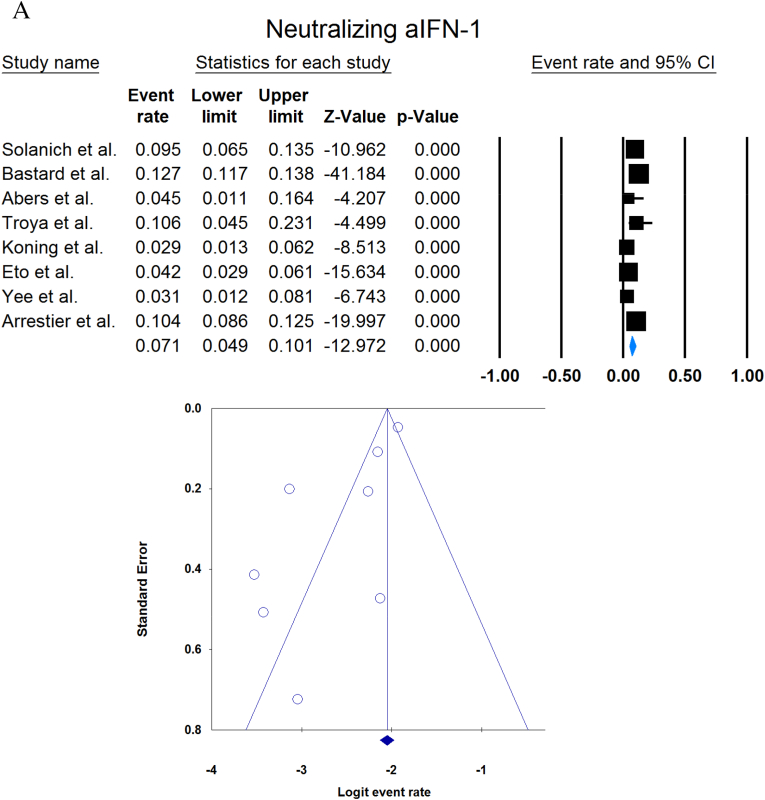

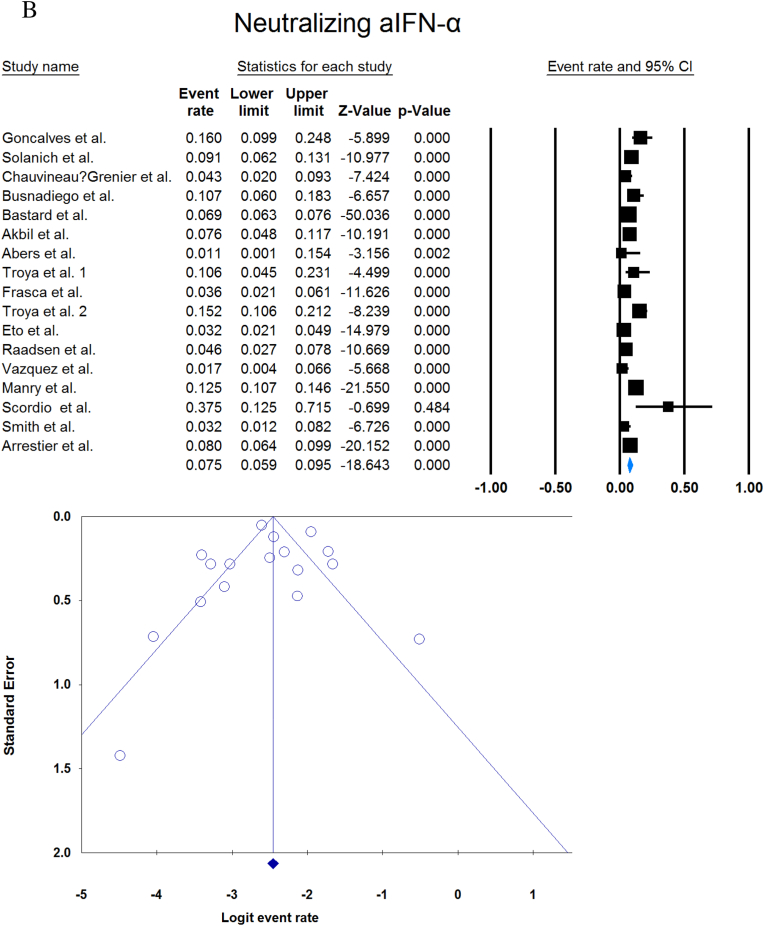

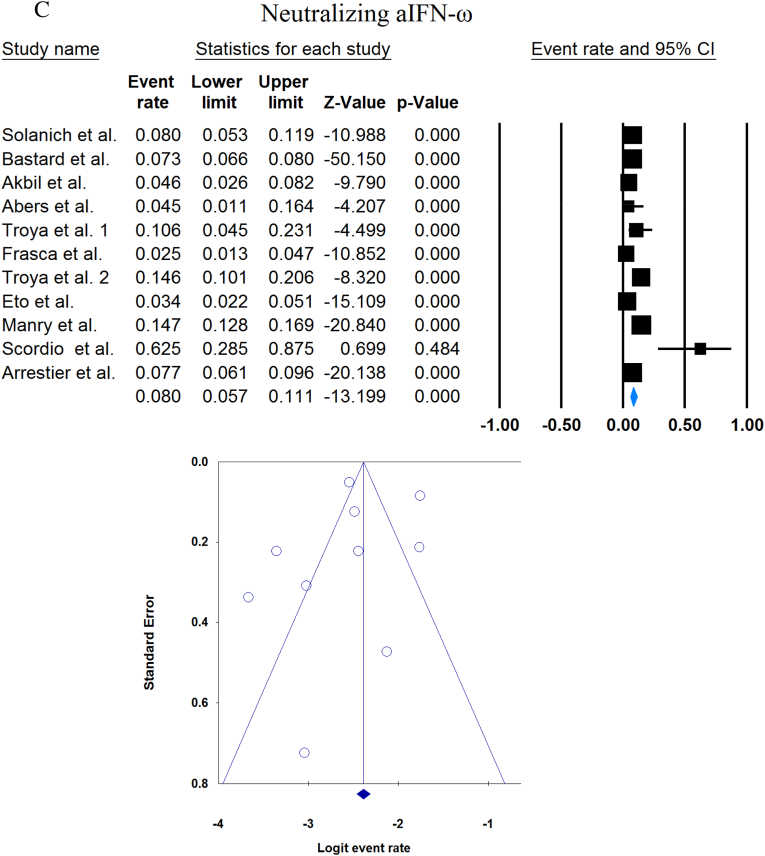

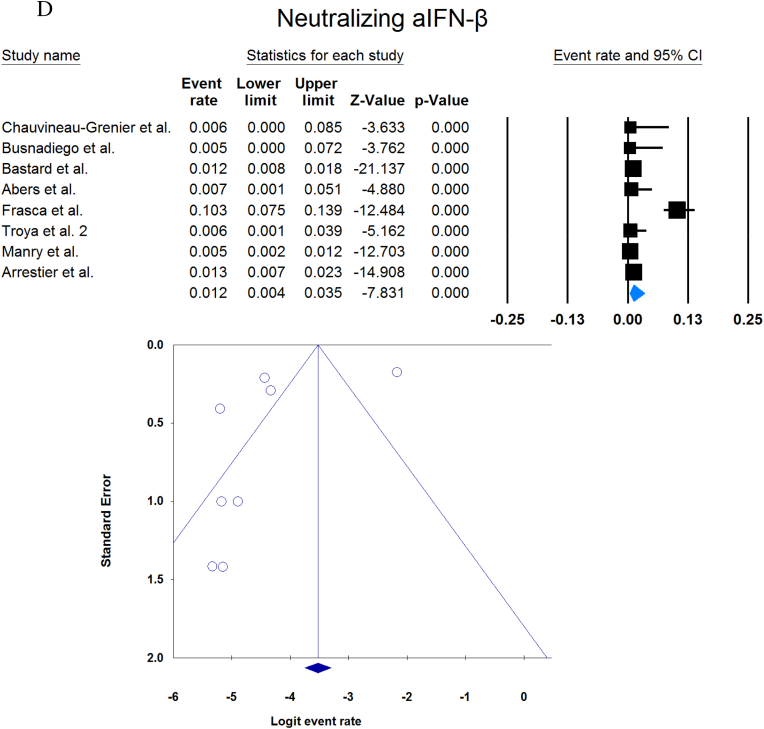
Overall prevalence of neutralizing aIFN-1 (A), aIFN-α (B), aIFN-ω (C), and aIFN-β (D) autoantibodies regardless of disease severity.

### 3.2 Moderate/mild versus critical/severe in terms of aIFN-1/α/ω

**Fig. 7:**
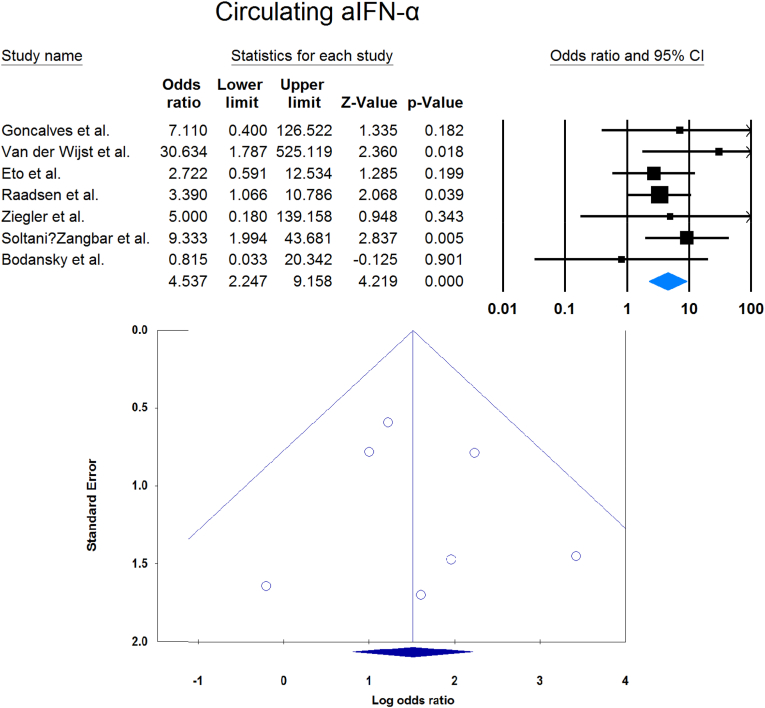
Circulating aIFN-α prevalence in critical/severe patients compared with moderate/mild patients.

**Fig. 8:**
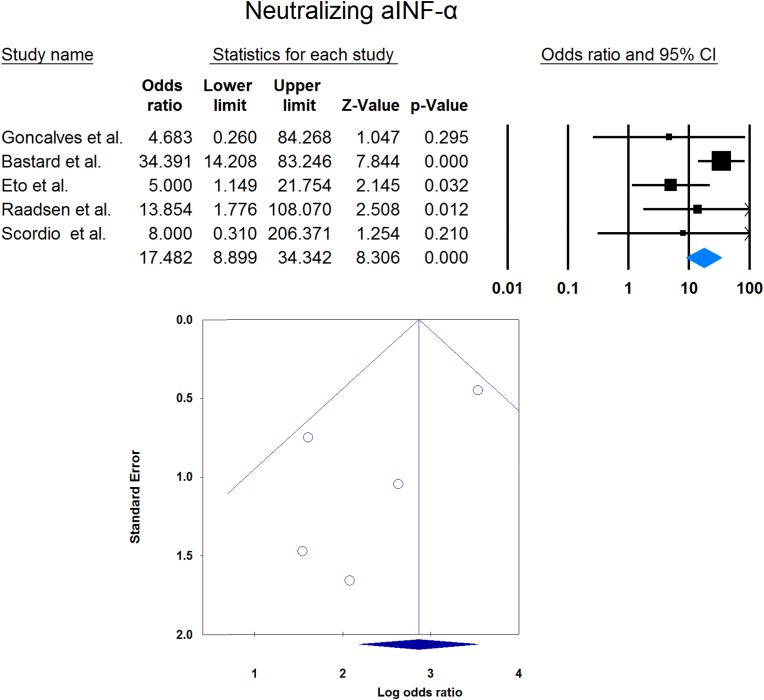
Neutralizing aIFN-α prevalence in critical/severe patients compared with moderate/mild patients.

**Fig. 9:**
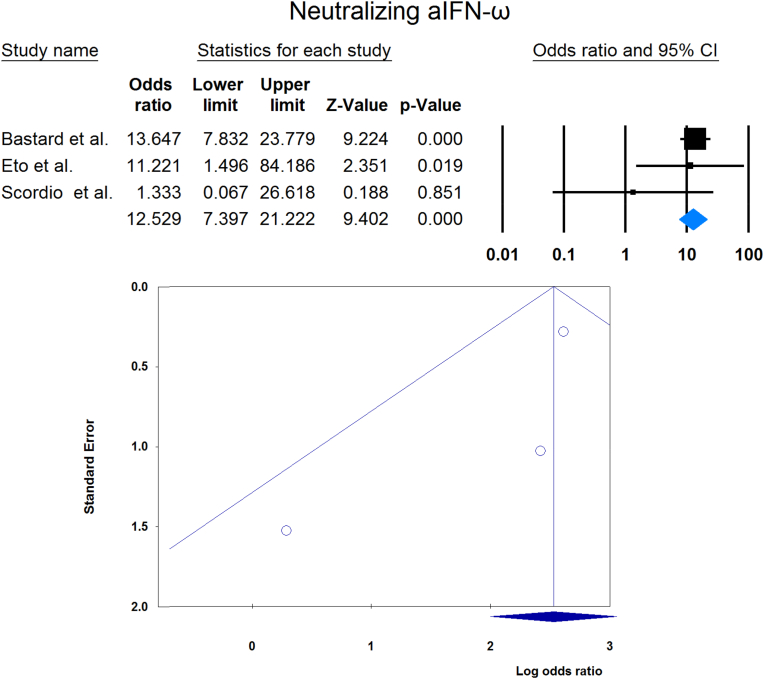
Circulating aIFN-ω prevalence in critical/severe patients compared with moderate/mild patients.

Meta-analysis showed that circulating aIFN-α was significantly more prevalent in critical/severe patients than in moderate/mild patients (OR = 4.537 [2.247, 9.158], p < 0.001, I2 = 0 %) (Fig. 7). Also, the prevalence of neutralizing aIFN-α was significantly higher in critical/severe patients than moderate/mild patients (OR = 17.482 [8.899, 34.342], p < 0.001, I2 = 34 %) (Fig. 8). The prevalence of neutralizing aIFN-ω was significantly higher in critical/severe than moderate/mild patients (OR = 12.529 [7.397, 21.222], p < 0.001, I2 = 11 %) (Fig. 9).

### 3.3 Qualitative report of comparison of plasma aIFN-α/ω levels

Soltani-Zangbar et al. reported higher aIFN-α2 levels in severe than mild patients [42], though three other studies showed no difference [33,47,48]. Three studies reported higher levels of aIFN-α2 among COVID-19 patients than in the control group [33,42,49], whereas two studies found no difference [47,48]. Furthermore, Chen et al. demonstrated higher aIFN-ω levels in severe patients than in mild patients [49], whereas Smith found no difference [47]. Only one study found no difference between aIFN-ω levels in COVID-19 patients and the control group [47]. Table 1 provides information on the characteristics of the studies reported.

### 3.4 Publication bias

Publication bias was assessed by visual inspection of the asymmetry of the funnel plot. The figures show no evidence of publication bias; however, for some subgroup analyses, there were insufficient studies to present proper funnel plots. Funnel plots are provided for all forest plots.

## 4 Discussion

This meta-analysis of 33 studies analyzed data from 13023 COVID-19 patients with respect to aIFN-1 AAbs. We estimated the pooled rate of seropositivity of circulating aIFN-1, aIFN-α, and aIFN-ω AAbs among COVID-19 patients to be approximately 17.8 % [13.8, 22.8], 7.2 % [4.7, 10.9], and 4.4 % [2.1, 8.6], and the rates of neutralizing aIFN-1, aIFN-α, aIFN-ω, and aIFN-β AAbs were 7.1 % [4.9, 10.1], 7.5 % [5.9, 9.5], 8.0 % [5.7, 11.1], and 1.2 % [0.4, 3.5], respectively. In contrast, the aIFN-α and aIFN-ω subtypes are present in about 0.3 % of the normal population [50]. The discrepancies in seropositivity rates reported by different studies can be attributed to the stage and severity of the disease, the measurement method and the characteristics of the patients. We performed a subgroup analysis in which patients were divided by COVID-19 disease severity that revealed significantly higher rates of aIFN-1, aIFN-α, and aIFN-ω in severe patients. A subsequent analysis provided further confirmation when patients were divided into severe/critical group or mild/moderate group, showed that the odds ratio of being positive for aIFN-α was significantly higher in the critical/severe group than in the mild/moderate group. One explanation for the high aIFN rate in severe patients is that IFN blockade provides an optimal environment for viral replication and disease progression [51]. Furthermore, patients with more severe disease are more prone to ICU admission and death, which we demonstrated that higher rates were associated with patients positive for aIFN-1 AAbs, though the association of IFN-1 AAbs with ICU admission and death may just be an epiphenomenon for disease severity. Our findings are supported by large cohorts and previous meta-analyses on this topic [50,52]. It has been suggested that aIFN-1, aIFN-α, and aIFN-ω AAbs may be appropriate biomarkers of disease severity and that their elimination by plasma exchange may improve clinical status [53]. However, IFN therapy has not been shown to improve mortality or length of hospital stay in COVID-19 patients [54]. Furthermore, length of hospital stay was not associated with aIFN-1 AAbs seropositivity, with no difference between positive and negative groups for IFN-α AAbs. However, the small size of the available sample may have led to a type 2 statistical error and a false negative result. The analysis also showed that aIFN-1 AAbs were more frequent in male subjects, which may explain previous findings of an increased incidence of severe disease in male subjects [55,56]. The recent meta-analysis by Wang et al. on this topic included 8 studies and concluded that AAbs against IFN-1 accumulate in 5 % of the total population and reach 10 % in severe patients. However, the prevalence of neutralizing and circulating AAbs was not reported separately [52]. In agreement with them, we found that aIFN-I AAbs were more prevalent in male subjects. Furthermore, although AAbs can decrease circulating levels of IFN-1 and its subtypes, a meta-analysis by Pires et al. showed that there were no significant differences in peripheral IFN-α in patients with different disease severity [57]. However, a significant difference was found between healthy control subjects and COVID -19 patients with mild disease.

Regarding the biomarker potential of aIFN AAbs, Beck et al. [58] suggested that an impaired IFN-1 response could be the hallmark of severe COVID-19. Zhang et al. also reported an association between SARS-CoV-2 infection and defects in genes regulating IFN-1 using extensive genetic sequencing [59]. There are two main hypotheses regarding the cause of an elevated aIFN-1. Firstly, AAbs facilitate viral replication because the host response system is neutralized by AAbs [19]. In support of this, Trouillet-Assant et al. [60] reported that a decreased plasma concentration of the IFN-1 cytokine predicts a poor prognosis and that patients with the lowest IFN-1 concentration have longer durations of admission. Secondly, viral evasion leads to the increased production of IFN-1, which contributes to the cytokine storm in COVID-19 disease [61], TNF-mediated inflammation [62], and high IFN-1 secretion impairs antibody production [63]. Massive production of IFN-1 leads to tissue damage, and autoantibodies may provide impaired protection against the cytokine storm [64]. In accordance with the second hypothesis, single-cell RNA-sequencing studies show hyperactivation of the IFN-1 pathway in severe patients, disrupting immune function, leading to overreaction and eventually resulting in organ damage [65,66]. Post-mortem analyzes of COVID-19 patients reveal a high concentration of IFN-1 in lung tissue [67]. Menezes et al. also demonstrated that higher levels of IFNB1 transcripts are found in the nasal mucosa of COVID-19 patients and predict a poor outcome [68].

IFN-1 are critical for controlling SARS-CoV-2 infection, which may explain the increased susceptibility of older adults with a reduced ability to secrete IFN-1 [59]. The Bodansky et al. study showed a low frequency of positive AAbs against IFN-α2, 0.4 % in children and adolescents [16], and sensitivity analysis on removing this young population in the Bodansky study showed a rate of 7.9 % [5.2, 11.8], indicating that aIFN-α2-AAbs mainly occur in older patients.

Our study had some limitations. Firstly, limited analysis of IFN-ω or aIFN-β levels could be performed as few studies reported them, increasing the risk of a false negative result. Secondly, another limitation is the low sensitivity of the assays used in some studies to measure aIFN-1 and its subtypes, which may have led to underreporting. Assessment of aIFN-1 in the post-COVID-19 phase may provide insights into the role of aIFN-1 AAbs in patients with long-COVID-19 and post-COVID-19 syndrome, and future studies should also investigate aIFN-γ AAbs [48].

## 5 Conclusion

In conclusion, aIFN-1 and its subtypes AAbs are associated with severe and critical COVID-19 disease and may be a predictive marker for a poor prognosis, particularly in men.

## Credit author statement

Conceptualization: AA, AS. Investigation: AA, AH, MA, MMM, ZS, TJ, AS. Writing - Original Draft: AA, AH, MA, NNN. Writing - Review & Editing: TJ, AS.

## Funding

No external fund was received for conducting this study.

## Declaration of competing interest

The authors declare that they have no known competing financial interests or personal relationships that could have appeared to influence the work reported in this paper.

## Data Availability

No data was used for the research described in the article.

## References

[1] Organization W.H. (2023).

[2] Galani I.-E., Rovina N., Lampropoulou V., Triantafyllia V., Manioudaki M., Pavlos E. (2021). Untuned antiviral immunity in COVID-19 revealed by temporal type I/III interferon patterns and flu comparison. Nat. Immunol..

[3] Tajbakhsh A., Gheibi Hayat S.M., Taghizadeh H., Akbari A., Inabadi M., Savardashtaki A. (2021). COVID-19 and cardiac injury: clinical manifestations, biomarkers, mechanisms, diagnosis, treatment, and follow up. Expert Rev. Anti-infect. Ther..

[4] Ghale R., Spottiswoode N., Anderson M.S., Mitchell A., Wang G., Calfee C.S. (2022). Prevalence of type-1 interferon autoantibodies in adults with non-COVID-19 acute respiratory failure. Respir. Res..

[5] Zhang Q., Pizzorno A., Miorin L., Bastard P., Gervais A., Le Voyer T. (2022). Autoantibodies against type I IFNs in patients with critical influenza pneumonia. J. Exp. Med..

[6] Abers M.S., Rosen L.B., Delmonte O.M., Shaw E., Bastard P., Imberti L. (2021). Neutralizing type-I interferon autoantibodies are associated with delayed viral clearance and intensive care unit admission in patients with COVID-19. Immunol. Cell Biol..

[7] Yee D., Tso M., Shaw E., Rosen L., Samuels E., Bastard P. (2021). Type I Interferon Autoantibodies Are Detected in Those with Critical COVID-19, Including a Young Female Patient.

[8] Bekisz J., Schmeisser H., Hernandez J., Goldman N.D., Zoon K.C. (2004). Mini ReviewHuman interferons alpha, beta and omega. Growth Factors.

[9] Goncalves D., Mezidi M., Bastard P., Perret M., Saker K., Fabien N. (2021). Antibodies against type I interferon: detection and association with severe clinical outcome in COVID-19 patients. Clinical & translational immunology.

[10] Frasca F., Scordio M., Santinelli L., Gabriele L., Gandini O., Criniti A. (2022). Anti-IFN-α/-ω neutralizing antibodies from COVID-19 patients correlate with downregulation of IFN response and laboratory biomarkers of disease severity. Eur. J. Immunol..

[11] Troya J., Bastard P., Casanova J.-L., Abel L., Pujol A. (2022). Low lymphocytes and IFN-neutralizing autoantibodies as biomarkers of COVID-19 mortality. J. Clin. Immunol..

[12] Casanova J.L., Anderson M.S. (2023). Unlocking life-threatening COVID-19 through two types of inborn errors of type I IFNs. J. Clin. Invest..

[13] Moher D., Liberati A., Tetzlaff J., Altman D.G. (2009). Preferred reporting items for systematic reviews and meta-analyses: the PRISMA statement. PLoS Med..

[14] Lemarquis A., Campbell T., Aranda-Guillén M., Hennings V., Brodin P., Kämpe O. (2021). Severe COVID-19 in an APS1 patient with interferon autoantibodies treated with plasmapheresis. J. Allergy Clin. Immunol..

[15] Moola S., Munn Z., Tufanaru C., Aromataris E., Sears K., Sfetcu R. (2017).

[16] Bodansky A., Vazquez S.E., Chou J., Novak T., Al-Musa A., Young C. (2023). NFKB2 haploinsufficiency identified via screening for IFN-α2 autoantibodies in children and adolescents hospitalized with SARS-CoV-2-related complications. J. Allergy Clin. Immunol..

[17] Wan X., Wang W., Liu J., Tong T. (2014). Estimating the sample mean and standard deviation from the sample size, median, range and/or interquartile range. BMC Med. Res. Methodol..

[18] Egger M., Davey Smith G., Schneider M., Minder C. (1997). Bias in meta-analysis detected by a simple, graphical test. BMJ (Clinical research ed).

[19] Bastard P., Rosen L.B., Zhang Q., Michailidis E., Hoffmann H.H., Zhang Y. (2020). Autoantibodies against type I IFNs in patients with life-threatening COVID-19. Science (New York, NY).

[20] Shaw E.R., Rosen L.B., Cheng A., Dobbs K., Delmonte O.M., Ferré E.M.N. (2022). Temporal dynamics of anti–type 1 interferon autoantibodies in patients with coronavirus disease 2019. Clin. Infect. Dis..

[21] van der Wijst M.G.P., Vazquez S.E., Hartoularos G.C., Bastard P., Grant T., Bueno R. (2021).

[22] Matuozzo D., Talouarn E., Marchal A., Zhang P., Manry J., Seeleuthner Y. (2023). Rare predicted loss-of-function variants of type I IFN immunity genes are associated with life-threatening COVID-19. Genome Med..

[23] Chauvineau-Grenier A., Bastard P., Servajean A., Gervais A., Rosain J., Jouanguy E. (2022). Autoantibodies neutralizing type I interferons in 20% of COVID-19 deaths in a French hospital. J. Clin. Immunol..

[24] Lopez J., Mommert M., Mouton W., Pizzorno A., Brengel-Pesce K., Mezidi M. (2021). Early nasal type I IFN immunity against SARS-CoV-2 is compromised in patients with autoantibodies against type I IFNs. J. Exp. Med..

[25] Manry J., Bastard P., Gervais A., Le Voyer T., Rosain J., Philippot Q. (2022). The risk of COVID-19 death is much greater and age dependent with type I IFN autoantibodies. Proc. Natl. Acad. Sci. USA.

[26] Carapito R., Li R., Helms J., Carapito C., Gujja S., Rolli V. (2022). Identification of driver genes for critical forms of COVID-19 in a deeply phenotyped young patient cohort. Sci. Transl. Med..

[27] Mathian A., Breillat P., Dorgham K., Bastard P., Charre C., Lhote R. (2022). Lower disease activity but higher risk of severe COVID-19 and herpes zoster in patients with systemic lupus erythematosus with pre-existing autoantibodies neutralising IFN-α. Ann. Rheum. Dis..

[28] Arrestier R., Bastard P., Belmondo T., Voiriot G., Urbina T., Luyt C.-E. (2022). Auto-antibodies against type I IFNs in > 10% of critically ill COVID-19 patients: a prospective multicentre study. Ann. Intensive Care.

[29] Solanich X., Rigo-Bonnin R., Gumucio V.D., Bastard P., Rosain J., Philippot Q. (2021). Pre-existing autoantibodies neutralizing high concentrations of type I interferons in almost 10% of COVID-19 patients admitted to intensive care in barcelona. J. Clin. Immunol..

[30] Troya J., Bastard P., Planas-Serra L., Ryan P., Ruiz M., de Carranza M. (2021). Neutralizing autoantibodies to type I IFNs in >10% of patients with severe COVID-19 pneumonia hospitalized in Madrid, Spain. J. Clin. Immunol..

[31] Scordio M., Frasca F., Santinelli L., Sorrentino L., Pierangeli A., Turriziani O. (2022). High frequency of neutralizing antibodies to type I Interferon in HIV-1 patients hospitalized for COVID-19. Clin. Immunol..

[32] Koning R., Bastard P., Casanova J.-L., Brouwer M.C., van de Beek D., van Agtmael M. (2021). Autoantibodies against type I interferons are associated with multi-organ failure in COVID-19 patients. Intensive Care Med..

[33] Raadsen M.P., Gharbharan A., Jordans C.C.E., Mykytyn A.Z., Lamers M.M., van den Doel P.B. (2022). Interferon-α2 auto-antibodies in convalescent plasma therapy for COVID-19. J. Clin. Immunol..

[34] Steels S., Van Elslande J., Cockx M., Frans G., Imbrechts M., Dillaerts D. (2022). Transient increase of pre-existing anti-IFN-α2 antibodies induced by SARS-CoV-2 infection. J. Clin. Immunol..

[35] Busnadiego I., Abela I.A., Frey P.M., Hofmaenner D.A., Scheier T.C., Schuepbach R.A. (2022). Critically ill COVID-19 patients with neutralizing autoantibodies against type I interferons have increased risk of herpesvirus disease. PLoS Biol..

[36] van der Wijst M.G.P., Vazquez S.E., Hartoularos G.C., Bastard P., Grant T., Bueno R. (2021). Type I interferon autoantibodies are associated with systemic immune alterations in patients with COVID-19. Sci. Transl. Med..

[37] Chang S.E., Feng A., Meng W., Apostolidis S.A., Mack E., Artandi M. (2021). New-onset IgG autoantibodies in hospitalized patients with COVID-19. Nat. Commun..

[38] Wang E.Y., Mao T., Klein J., Dai Y., Huck J.D., Jaycox J.R. (2021). Diverse functional autoantibodies in patients with COVID-19. Nature.

[39] Vazquez S.E., Bastard P., Kelly K., Gervais A., Norris P.J., Dumont L.J. (2021). Neutralizing autoantibodies to type I interferons in COVID-19 convalescent donor plasma. J. Clin. Immunol..

[40] Ziegler C.G.K., Miao V.N., Owings A.H., Navia A.W., Tang Y., Bromley J.D. (2021). Impaired local intrinsic immunity to SARS-CoV-2 infection in severe COVID-19. Cell.

[41] Acosta-Ampudia Y., Monsalve D.M., Rojas M., Rodríguez Y., Gallo J.E., Salazar-Uribe J.C. (2021). COVID-19 convalescent plasma composition and immunological effects in severe patients. J. Autoimmun..

[42] Soltani-Zangbar M.S., Parhizkar F., Ghaedi E., Tarbiat A., Motavalli R., Alizadegan A. (2022). A comprehensive evaluation of the immune system response and type-I Interferon signaling pathway in hospitalized COVID-19 patients. Cell Commun. Signal..

[43] Savvateeva E., Filippova M., Valuev-Elliston V., Nuralieva N., Yukina M., Troshina E. (2021). Microarray-based detection of antibodies against SARS-CoV-2 proteins, common respiratory viruses and type I interferons. Viruses.

[44] Eto S., Nukui Y., Tsumura M., Nakagama Y., Kashimada K., Mizoguchi Y. (2022). Neutralizing type I interferon autoantibodies in Japanese patients with severe COVID-19. J. Clin. Immunol..

[45] Akbil B., Meyer T., Stubbemann P., Thibeault C., Staudacher O., Niemeyer D. (2022). Early and rapid identification of COVID-19 patients with neutralizing type I interferon auto-antibodies. J. Clin. Immunol..

[46] Bastard P., Gervais A., Le Voyer T., Rosain J., Philippot Q., Manry J. (2021). Autoantibodies neutralizing type I IFNs are present in ∼4% of uninfected individuals over 70 years old and account for ∼20% of COVID-19 deaths. Science immunology.

[47] Smith N., Possémé C., Bondet V., Sugrue J., Townsend L., Charbit B. (2022). Defective activation and regulation of type I interferon immunity is associated with increasing COVID-19 severity. Nat. Commun..

[48] Chen P.K., Yeo K.J., Chang S.H., Liao T.L., Chou C.H., Lan J.L. (2023). The detectable anti-interferon-γ autoantibodies in COVID-19 patients may be associated with disease severity. Virol. J..

[49] Simula E.R., Manca M.A., Noli M., Jasemi S., Ruberto S., Uzzau S. (2022). Increased presence of antibodies against type I interferons and human endogenous retrovirus W in intensive care unit COVID-19 patients. Microbiol. Spectr..

[50] Bastard P., Gervais A., Le Voyer T., Rosain J., Philippot Q., Manry J. (2021). Autoantibodies neutralizing type I IFNs are present in ∼4% of uninfected individuals over 70 years old and account for ∼20% of COVID-19 deaths. Science immunology.

[51] Fajgenbaum D.C., Hayday A.C., Rogers A.J., Towers G.J., Wack A., Zanoni I. (2022). Anti-type I interferon antibodies as a cause of severe COVID-19. Faculty reviews.

[52] Wang X., Tang Q., Li H., Jiang H., Xu J., Bergquist R. (2023). Autoantibodies against type I interferons in COVID-19 infection: a systematic review and meta-analysis. Int. J. Infect. Dis..

[53] de Prost N., Bastard P., Arrestier R., Fourati S., Mahévas M., Burrel S. (2021). Plasma exchange to rescue patients with autoantibodies against type I interferons and life-threatening COVID-19 pneumonia. J. Clin. Immunol..

[54] Akbari A., Razmi M., Sedaghat A., Alavi Dana S.M.M., Amiri M., Halvani A.M. (2022). Comparative effectiveness of pharmacological interventions on mortality and the average length of hospital stay of patients with COVID-19: a systematic review and meta-analysis of randomized controlled trials. Expert Rev. Anti-infect. Ther..

[55] Akbari A., Fathabadi A., Razmi M., Zarifian A., Amiri M., Ghodsi A. (2022). Characteristics, risk factors, and outcomes associated with readmission in COVID-19 patients: a systematic review and meta-analysis. Am. J. Emerg. Med..

[56] Peckham H., de Gruijter N.M., Raine C., Radziszewska A., Ciurtin C., Wedderburn L.R. (2020). Male sex identified by global COVID-19 meta-analysis as a risk factor for death and ITU admission. Nat. Commun..

[57] da Silva R.P., Gonçalves J.I.B., Zanin R.F., Schuch F.B., de Souza A.P.D. (2021). Circulating type I interferon levels and COVID-19 severity: a systematic review and meta-analysis. Front. Immunol..

[58] Beck D.B., Aksentijevich I. (2020). Susceptibility to severe COVID-19. Science (New York, NY).

[59] Zhang Q., Bastard P., Liu Z., Le Pen J., Moncada-Velez M., Chen J. (2020). Inborn errors of type I IFN immunity in patients with life-threatening COVID-19. Science (New York, NY).

[60] Trouillet-Assant S., Viel S., Gaymard A., Pons S., Richard J.C., Perret M. (2020). Type I IFN immunoprofiling in COVID-19 patients. J. Allergy Clin. Immunol..

[61] Nile S.H., Nile A., Qiu J., Li L., Jia X., Kai G. (2020). COVID-19: pathogenesis, cytokine storm and therapeutic potential of interferons. Cytokine Growth Factor Rev..

[62] Huys L., Van Hauwermeiren F., Dejager L., Dejonckheere E., Lienenklaus S., Weiss S. (2009). Type I interferon drives tumor necrosis factor-induced lethal shock. J. Exp. Med..

[63] Costa Silva R.C.M., Bandeira-Melo C., Paula Neto H.A., Vale A.M., Travassos L.H. (2022). COVID-19 diverse outcomes: aggravated reinfection, type I interferons and antibodies. Med. Hypotheses.

[64] Ku C.-L., Chi C.-Y., von Bernuth H., Doffinger R. (2020). Autoantibodies against cytokines: phenocopies of primary immunodeficiencies?. Hum. Genet..

[65] Zhang J.-Y., Wang X.-M., Xing X., Xu Z., Zhang C., Song J.-W. (2020). Single-cell landscape of immunological responses in patients with COVID-19. Nat. Immunol..

[66] Lulin H., Yi S., Bo G., Li J., Xiaoqi L., Jialiang Y. (2020). Blood single cell immune profiling reveals the interferon-MAPK pathway mediated adaptive immune response for COVID-19. medRxiv.

[67] Lee J.S., Park S., Jeong H.W., Ahn J.Y., Choi S.J., Lee H. (2020). Immunophenotyping of COVID-19 and influenza highlights the role of type I interferons in development of severe COVID-19. Science immunology.

[68] Menezes S.M., Braz M., Llorens-Rico V., Wauters J., Van Weyenbergh J. (2021). Endogenous IFNβ expression predicts outcome in critical patients with COVID-19. The Lancet Microbe.

