# Supplementary material for: Role of autoantibodies targeting interferon type 1 in COVID-19 severity: A systematic review and meta-analysis
Source: J Transl Autoimmun. 2023 Oct 14;7:100219. doi: 10.1016/j.jtauto.2023.100219 (PMC10587724; doi:10.1016/j.jtauto.2023.100219)
Supplement: Multimedia component 1 [file mmc1.docx]

**Supplementary file**

|  | **Table S1: Search Strategy** | |
| --- | --- | --- |
|  | **Pubmed (May 9, 2023)** | |
| **Results** | **Query** | **Search** |
| 348,270 | ("severe acute respiratory syndrome coronavirus 2"[title/abstract] OR "Wuhan coronavirus"[title/abstract] OR "Wuhan seafood market pneumonia virus"[title/abstract] OR "COVID19 virus"[title/abstract] OR "COVID-19 virus"[title/abstract] OR "coronavirus disease 2019 virus"[title/abstract] OR "SARS-CoV-2"[title/abstract] OR "SARS2"[title/abstract] OR "2019-nCoV"[title/abstract] OR "2019 novel coronavirus"[title/abstract] OR "COVID-19"[title/abstract] OR "2019 novel coronavirus infection"[title/abstract] OR "COVID19"[title/abstract] OR "coronavirus disease 2019"[title/abstract] OR "coronavirus disease-19"[title/abstract] OR "2019-nCoV disease"[title/abstract] OR "2019 novel coronavirus disease"[title/abstract] OR "2019-nCoV infection"[title/abstract] OR "Coronavirus Infections"[title/abstract] OR "Coronavirus Infection"[title/abstract] OR "Infection, Coronavirus"[title/abstract] OR "Infections, Coronavirus"[title/abstract] OR "novel coronavirus"[title/abstract] OR "Covid*"[title/abstract] OR "sars 2"[title/abstract]) | **#1** (COVID-19) |
| 74,394 | ((auto-immunity[title/abstract] OR autoimmunity[title/abstract]) AND (antibod*[title/abstract]) OR Auto-antibod*[title/abstract] OR Autoantibod*[title/abstract] OR "Anti-Interferon*"[title/abstract] OR Anti-IFN*[title/abstract] OR "Antibodies against type I interferon"[title/abstract] OR "Neutralizing Type I Interferon*"[Title/Abstract]) | **#2** (Interferon) |
| **869** | **#1 AND #2** | **Final** |
|  | **Scopus (May 10, 2023)** | |
| 520,821 | TITLE-ABS-KEY("severe acute respiratory syndrome coronavirus 2" OR "Wuhan coronavirus" OR "Wuhan seafood market pneumonia virus" OR "COVID19 virus" OR "COVID-19 virus" OR "coronavirus disease 2019 virus" OR "SARS-CoV-2" OR "SARS2" OR "2019-nCoV" OR "2019 novel coronavirus" OR "COVID-19" OR "2019 novel coronavirus infection" OR "COVID19" OR "coronavirus disease 2019" OR "coronavirus disease-19" OR "2019-nCoV disease" OR "2019 novel coronavirus disease" OR "2019-nCoV infection" OR "Coronavirus Infections" OR "Coronavirus Infection" OR "Infection, Coronavirus" OR "Infections, Coronavirus" OR "novel coronavirus" OR "Covid*" OR "sars 2") | **#1** (COVID-19) |
| 142,552 | TITLE-ABS-KEY ( ( ( "auto-immunity" OR autoimmunity ) AND "antibod*" ) OR "Auto-antibod*" OR "Autoantibod*" OR "Anti-Interferon*" OR "Anti-IFN*" OR "Antibodies against type I interferon" OR "Neutralizing Type I Interferon*") | **#2** (Interferon) |
| **1536** | **#1 AND #2** | **Final** |
|  | **Embase (May 10, 2023)** | |
| 382,837 | ("severe acute respiratory syndrome coronavirus 2":ti,ab OR "Wuhan coronavirus":ti,ab OR "Wuhan seafood market pneumonia virus":ti,ab OR "COVID19 virus":ti,ab OR "COVID-19 virus":ti,ab OR "coronavirus disease 2019 virus":ti,ab OR "SARS-CoV-2":ti,ab OR "SARS2":ti,ab OR "2019-nCoV":ti,ab OR "2019 novel coronavirus":ti,ab OR "COVID-19":ti,ab OR "2019 novel coronavirus infection":ti,ab OR "COVID19":ti,ab OR "coronavirus disease 2019":ti,ab OR "coronavirus disease-19":ti,ab OR "2019-nCoV disease":ti,ab OR "2019 novel coronavirus disease":ti,ab OR "2019-nCoV infection":ti,ab OR "Coronavirus Infections":ti,ab OR "Coronavirus Infection":ti,ab OR "Infection, Coronavirus":ti,ab OR "Infections, Coronavirus":ti,ab OR "novel coronavirus":ti,ab OR "Covid*":ti,ab OR "sars 2":ti,ab) | **#1** (COVID-19) |
| 108,428 | (((auto-immunity:ti,ab OR autoimmunity:ti,ab) AND antibod*:ti,ab) OR Auto-antibod*:ti,ab OR Autoantibod*:ti,ab OR "Anti-Interferon*":ti,ab OR Anti-IFN*:ti,ab OR "Antibodies against type I interferon":ti,ab OR "Neutralizing Type I Interferon*":ti,ab) | **#2** (Interferon) |
| **1153** | **#1 AND #2** | **Final** |
|  | **Web Of Science (December 2, 2022)** | |
| 358,022 | ((TI=("severe acute respiratory syndrome coronavirus 2" OR "Wuhan coronavirus" OR "Wuhan seafood market pneumonia virus" OR "COVID19 virus" OR "COVID-19 virus" OR "coronavirus disease 2019 virus" OR "SARS-CoV-2" OR "SARS2" OR "2019-nCoV" OR "2019 novel coronavirus" OR "COVID-19" OR "2019 novel coronavirus infection" OR "COVID19" OR "coronavirus disease 2019" OR "coronavirus disease-19" OR "2019-nCoV disease" OR "2019 novel coronavirus disease" OR "2019-nCoV infection" OR "Coronavirus Infections" OR "Coronavirus Infection" OR "Infection, Coronavirus" OR "Infections, Coronavirus" OR "novel coronavirus" OR Covid* OR "sars 2" ) OR AB=("severe acute respiratory syndrome coronavirus 2" OR "Wuhan coronavirus" OR "Wuhan seafood market pneumonia virus" OR "COVID19 virus" OR "COVID-19 virus" OR "coronavirus disease 2019 virus" OR "SARS-CoV-2" OR "SARS2" OR "2019-nCoV" OR "2019 novel coronavirus" OR "COVID-19" OR "2019 novel coronavirus infection" OR "COVID19" OR "coronavirus disease 2019" OR "coronavirus disease-19" OR "2019-nCoV disease" OR "2019 novel coronavirus disease" OR "2019-nCoV infection" OR "Coronavirus Infections" OR "Coronavirus Infection" OR "Infection, Coronavirus" OR "Infections, Coronavirus" OR "novel coronavirus" OR "Covid*" OR "sars 2" ))) | **#1** (COVID-19) |
| 67,284 | (TI=((("auto-immunity" OR autoimmunity) AND "antibod*") OR "Auto-antibod*" OR "Autoantibod*" OR "Anti-Interferon*" OR "Anti-IFN*" OR "Antibodies against type I interferon" OR "Neutralizing Type I Interferon*") OR AB=((("auto-immunity" OR autoimmunity) AND "antibod*") OR "Auto-antibod*" OR "Autoantibod*" OR "Anti-Interferon*" OR "Anti-IFN*" OR "Antibodies against type I interferon" OR "Neutralizing Type I Interferon*")) | **#2** (Interferon) |
| **592** | **#1 AND #2** | **Final** |

**Supplementary Table S****2A**: Quality assessment table for cohort studies based on JBI Critical Appraisal

| **Study** | **1. Were the two groups similar and recruited from the same population?** | **2. Were the exposures measured similarly to assign people to both exposed and unexposed groups?** | **3. Was the exposure measured in a valid and reliable way?** | **4.Were confounding factors identified?** | **5. Were strategies to deal with confounding factors stated?** | **6. Were the groups/participants free of the outcome at the start of the study (or at the moment of exposure)?** | **7. Were the outcomes measured in a valid and reliable way?** | **8. Was the follow up time reported and sufficient to be long enough for outcomes to occur?** | **9. Was follow up complete, and if not, were the reasons to loss to follow up described and explored?** | **10. Were strategies to address incomplete follow up utilized?** | **11. Was appropriate statistical analysis used?** |
| --- | --- | --- | --- | --- | --- | --- | --- | --- | --- | --- | --- |
| **Goncalves et al.** | Y | Y | Y | N | N | Y | Y | N | Y | N | Y |
| **Van der Wijst et al.** | Y | Y | Y | N | N | U | Y | Y | Y | N | Y |
| **Solanich et al.** | Y | Y | Y | N | N | Y | Y | Y | Y | N | Y |
| **Chauvineau-Grenier et al.** | Y | Y | N | N | N | Y | Y | Y | Y | N | Y |
| **Busnadiego et al.** | Y | Y | Y | N | N | U | Y | Y | Y | N | U |
| **Abers et al.** | Y | U | U | N | N | Y | Y | Y | Y | N | Y |
| **Troya et al.** | Y | Y | Y | N | N | Y | Y | Y | Y | N | Y |
| **Akbil et al.** | N | Y | Y | N | N | Y | Y | Y | Y | N | Y |
| **Lopez et al.** | U | Y | Y | N | N | U | Y | Y | Y | N | Y |
| **Bastard et al. 2020** | N | N | N | N | N | Y | Y | Y | Y | N | Y |
| **Bastard et al. 2021** | N | N | N | N | N | Y | Y | Y | Y | N | Y |
| **Frasca et al.** | Y | Y | N | N | N | Y | Y | Y | Y | N | Y |
| **Troya et al.** | Y | U | U | N | N | Y | U | Y | Y | N | Y |
| **Koning et al.** | Y | Y | Y | N | N | Y | Y | Y | Y | N | Y |
| **Eto et al.** | Y | Y | N | N | N | Y | Y | Y | Y | N | Y |
| **Raadsen et al.** | Y | Y | Y | N | N | Y | Y | Y | Y | N | Y |
| **Chang et al.** | N | Y | Y | N | N | U | Y | Y | Y | N | Y |
| **Wang et al.** | Y | Y | Y | N | N | Y | Y | Y | Y | N | Y |
| **Vazquez et al.** | Y | Y | Y | N | N | U | Y | Y | Y | N | U |
| **Manry et al.** | N | N | N | N | N | Y | Y | Y | Y | N | Y |
| **Savvateeva et al.** | U | Y | Y | N | N | U | Y | Y | Y | N | Y |
| **Acosta-Ampudia et al.** | N | Y | Y | Y | Y | Y | Y | Y | Y | N | Y |
| **Carapito et al.** | Y | Y | Y | Y | Y | Y | Y | Y | Y | N | Y |
| **Yee et al.** | Y | Y | U | N | N | U | U | U | U | N | U |
| **Steels et al.** | Y | Y | Y | Y | Y | Y | Y | Y | Y | N | Y |
| **Ziegler et al.** | Y | Y | Y | Y | Y | Y | Y | Y | Y | N | Y |
| **Scordio et al.** | Y | Y | Y | N | N | Y | Y | U | Y | N | U |
| **Mathian et al.** | Y | Y | U | N | N | Y | Y | U | U | N | Y |
| **Smith et al.** | Y | Y | U | N | N | Y | Y | U | U | N | Y |
| **Arrestier et al.** | Y | Y | Y | N | N | Y | Y | Y | Y | N | Y |
| **Bodansky et al.** | Y | Y | Y | Y | Y | Y | Y | Y | Y | N | Y |

Y: Yes, U:Unclear, N: No

**Supplementary Table S2B**: Quality assessment table for cross-sectional studies based on JBI Critical Appraisal

| **Study** | **Were the criteria for inclusion in the sample clearly defined?** | **Were the study subjects and the setting described in detail?** | **Was the exposure measured in a valid and reliable way?** | **Were objective, standard criteria used for measurement of the condition?** | **Were confounding factors identified?** | **Were strategies to deal with confounding factors stated?** | **Were the outcomes measured in a valid and reliable way?** | **Was appropriate statistical analysis used?** |
| --- | --- | --- | --- | --- | --- | --- | --- | --- |
| **Soltani‑Zangbar et al.** | Y | Y | Y | Y | Y | Y | Y | Y |
